# Combination of Interleukin-27 and MicroRNA for Enhancing Expression of Anti-Inflammatory and Proosteogenic Genes

**DOI:** 10.1155/2017/6365857

**Published:** 2017-02-07

**Authors:** Manoel Figueiredo Neto, Marxa L. Figueiredo

**Affiliations:** Department of Basic Medical Sciences, Purdue University College of Veterinary Medicine, West Lafayette, IN 47907, USA

## Abstract

Remission of inflammation has become an achievable goal in inflammatory or rheumatoid arthritis (RA); however, bone erosion continues in many patients. Interleukin- (IL-) 27 regulates immune and bone cell balance and also suppresses activities of several inflammatory cell types in RA. Despite its promise, challenges to clinical translation of IL-27 have been its partial effects* in vivo*. Due to their ability to modulate plasticity of bone and immune cell differentiation, we examined the potential for several microRNA (miR) candidates in enhancing the effects of IL-27. Using differentiation, luciferase, and real time quantitative PCR assays, we show that IL-27 promotes osteoblast differentiation, reduces expression of osteoblast inhibitory genes, and reduces osteoclast differentiation, and results suggest a potential coordination with TGF*β*/BMP/SMAD and JAK/STAT pathways. We selected miRNA regulators of these and related pathways to examine whether the effects of IL-27 could be augmented for therapeutic applications. miR-29b and miR-21 augmented IL-27 proosteogenic while downregulating osteoclastogenic signals and also worked to reduce inflammatory signaling in activated macrophages, while miR-21 and miR-20b worked with IL-27 to reduce inflammatory gene expression in fibroblasts and T cells. It appears that several miRNAs can be utilized to enhance IL-27's impact on modulating osteogenesis and reducing proinflammatory signaling.

## 1. Introduction

There is a complex network of cytokines and receptors signaling in inflammatory diseases including inflammatory or rheumatoid arthritis (RA) and osteoarthritis. In RA, chronic inflammatory synovitis and progressive joint destruction result in severe disability and increased incidence of patient mortality. Autoreactive T cells and inflammatory cytokines such as tumor necrosis factor (TNF), interleukin- (IL-) 6, and IL-17a play a key role in the pathology of inflammatory arthritis. These cells and cytokines also induce the production of matrix metalloproteinases (MMP) as well as the maturation and activation of osteoclasts, which promote destruction of cartilage and bone, respectively. It is now known that significant progress can be made in treating joint damage in the early stages of the disease, that is, prior to joint destruction. The combined use of methotrexate, a synthetic disease-modifying antirheumatic drug, and biologics targeting TNF and interleukin- (IL-) 6 has revolutionized the treatment of RA. Clinical remission of inflammation has become an achievable goal in many patients. However, treating inflammation alone is not enough, as bone erosion continues in many patients experiencing clinical remission [1]. Thus the goals of maintaining clinical remission but also inhibiting structural changes are now important long-term treatment outcomes for RA.

Current data points to a novel use for cytokine interleukin-27 (IL-27) as a regulator of immune and bone cell balance. IL-27 is a heterodimeric cytokine composed of the subunits p28 and EBI3, which binds to the heterodimeric WSX1/gp130 receptor. Mechanisms of action of IL-27 on bone include the ability to naturally antagonize IL-6 [2] by virtue of its competition for the common receptor subunit gp130. IL-27 has a direct effect on bone cells, reducing osteoclastogenesis and promoting osteoblast proliferation and maturation [3, 4]. These effects are mediated via redirecting signaling from Signal Transducer and Activator of Transcription (Stat) 3 to Stat1 in bone cells and reducing receptor activator of nuclear factor kappa-B ligand (RANKL) expression in osteoclasts. Indirect effects are through altering the balance of CD4 T cells towards an anti-inflammatory profile (Treg > Th17) [5]. Also, IL-27 suppresses activities of several immune and synovial cells mediating the onset and maintenance of inflammation [6]. A recent report used subcutaneous delivery of Fc-IL27 to confirm its high promise for controlling collagen-induced arthritis through reducing Th17 proinflammatory cells without systemic toxicity [5]. However, Fc-IL27 was a partially effective system, with arthritis incidence reductions of ~50% and Th17 reductions of ~40% [5]. IL-27 inhibited production of IL-17 through downregulation of ROR*γτ* and STAT3 and upregulated Foxp3 and IL-10. In vitro differentiated Treg from human peripheral blood mononuclear cells of either healthy or RA patients were stronger suppressors of effector T cell proliferation in the presence of IL-27 [5]; thus this cytokine has high potential for controlling inflammation. IL-27 has anti-inflammatory effects on human and mouse myeloid cells through downregulation of TNFR and IL-1R and by production of IL-10 [7, 8] and downregulates several proinflammatory or osteoclastogenic signaling axes, including IL-6, TNF, IL1b, MMPs, and GM-CSF [3]. For repairing bone, our previous work [3, 4] shows that IL-27 can partially promote repair of prostate tumor-induced bone lesions in vivo. IL-27 treatment restores and maintains bone density similar to that of “no lesion” control throughout the 4-week study. Despite its promise, this gene delivery approach was effective only in repairing ~50% of limbs examined (correlated with serum levels achieved), as the vector was transiently expressed (<30 days), limiting long-term applications for treating bone erosions. Thus new strategies need to be explored for efficiently optimizing the effects of IL-27 in treating inflammation and repairing bone erosions.

The exciting recent findings that microRNA (miRNA) can regulate T cell and bone cell differentiation by modifying their phenotypic plasticity has opened up the possibility of using these epigenetic modifiers as therapeutics. miRNAs are short (~19–23 nt), single-stranded noncoding RNA molecules that affect the stability of messenger RNA and in some cases influence protein translation through partial sequence complementation with their interacting mRNA targets. For therapeutic purposes, synthetic* miRNA mimics* can be used that have the same function as an endogenous mature miRNA. miRNA mimics thus can be used for rebalancing inflammatory and bone cell phenotypes in conditions such as RA [9], promoting osteoblast differentiation, and inhibiting osteoclast activity. Based on the literature, several miRNA candidates can modify the plasticity of bone and immune cells. Based on the literature, we thus selected a panel of miRNAs to examine their ability to augment the effects of IL-27 on proinflammatory and/or proosteogenic genes. In this report we will show several miRNAs with multifunctional activity on synovial, bone, or immune cells that should be excellent candidates for augmenting the effects of IL-27 on bone repair.

## 2. Materials and Methods

### 2.1. Cell Culture and Differentiation Experiments

MC-3T3-E1 clone 14 mouse preosteoblasts were obtained from American Type Culture Collection (ATCC, Manassas, VA) and cultured in 10% heat-inactivated FBS/alpha-MEM (Invitrogen, Grand Island, NY) media with 1x antibiotic-antimycotic (ThermoFisher, Gibco, Waltham, MA). Heat-inactivation of FBS (ATCC) was carried out at 55°C for 30 min followed by storage at 4°C prior to addition to media. RAW 264.7 mouse monocytic cells were obtained from ATCC cultured in DMEM/10% FBS with 1% PS and gently scraped for passaging. NIH3T3 mouse fibroblasts were obtained from ATCC and cultured in 10% FBS/DMEM (Mediatech, Manassas, VA) with 1x antibiotic-antimycotic. EL4 murine T cells were obtained from ATCC and grown on DMEM (Mediatech) with 10% horse serum and 1x antibiotic-antimycotic and Jurkat human T cells were obtained from ATCC and grown on RPMI-1640 (Mediatech) and 10% FBS with 1x antibiotic-antimycotic. For bone cell differentiation assays, 1–1.5 × 10^5^ cells were seeded in 12-well format in triplicate and cultured with differentiation supplements as per manufacturer's instructions using supplements for differentiation. For inducing osteoblast MC3T3-E1(14) differentiation, 50 *µ*g/ml ascorbic acid and 10 mM *β*-glycerol phosphate were used when cells reached confluence in a 24-well plate format using 0.2% heat-inactivated FBS (ATCC). Cells were assayed at day 21 after supplement treatment. Following culturing in differentiation supplements, cells were fixed in iced cold 70% ethanol for 1 h at room temperature and stained for mineralization (mature osteoblasts) using Alizarin S Red (Millipore, Billerica, MA) with manufacturer's protocols. For inducing RAW264.7 cell fusion and differentiation into mature multinucleated cells (MNC) or osteoclasts, we cultured cells for 6 days in 35 ng/ml RANKL and stained them for detection of the marker protein tartrate-resistant acid phosphatase (TRAP) (Sigma-Aldrich, St. Louis, MO) as described [3]. Multinucleated cells were counted in 5–10 independent high power microscopic fields and expressed as an average of MNC/field.

### 2.2. Vectors

Plasmid DNA vectors were prepared as described in [3] using GeneJet endotoxin free kits (Fisher). For luciferase assays, constructs responsive to the active (phosphorylated) form of STAT1 or STAT3 were used (STAT1.GAS/ISRE- and STAT3/3-Luc; Panomics, Fremont, CA) or OPN-, RUNX2-, IL-17a-, TRAF2-, and TLR10-Luc (Switchgear Genomics, Carlsbad, CA) and Tcf/Lef-, BRE-, and SBE/FBE-Luc (SBE4-) or TNF-Luc (12456, 45126, 16525, and 11110, Addgene, Cambridge, MA) and 2% CMV-Bgal (Clontech, Mountain View, CA) as a transfection control to transfect cells using Lipofectamine 3000 or DMRIE-C reagents (Invitrogen, Carlsbad, CA) according to manufacturer's protocols [10]. miRNA mimics were purchased from Sigma-Aldrich at 5 nmol and utilized in a transfection at a 5 pmol final concentration. miRNA sequences are 100% conserved in human and mouse miRNA according to the miRbase database [11] and are as follows, miR-17, MIMAT0000070,* CAAAGUGCUUACAGUGCAGGUAG*, miR-210, MIMAT0000267,* CUGUGCGUGUGACAGCGGCUGA*, miR-29b-3p, MIMAT0000100, *UAGCACCAUUUGAAAUCAGUGUU,* miR-20b, MIMAT0001413,* CAAAGUGCUCAUAGUGCAGGUAG*, miR-10a, MIMAT0000253,* UACCCUGUAGAUCCGAAUUUGUG*, miR-let7f-5p, MIMAT0000067, *UGAGGUAGUAGAUUGUAUAGUU,* and miR-21-5p, MIMAT0000076,* UAGCUUAUCAGACUGAUGUUGA*.

### 2.3. Luciferase Reporter Assays and Transfections

For predifferentiating cells prior to 48 h transfections, osteoblasts were prepared by culture of MC3T3-E1(14) in 50 *µ*g/ml ascorbic acid and 10 mM *β*-glycerol phosphate in 96 wells for 5 days and osteoclasts prepared by culture of RAW264.7 cells in 35 ng/ml RANKL for 4 days. Then, these bone cells were transfected with plasmid DNA plus miRNA using Lipofectamine 3000 and allowed to express the luciferase vectors for 48 h prior to collection (day 7 for differentiating osteoblasts and day 6 for osteoclasts). For cell activation, macrophages and NIH3T3 received 0.15 *μ*g plasmid DNA and 5 pmol miRNA (0.24 *μ*g) using Lipofectamine 3000 for 6 h; then medium was changed to 10% FBS-containing medium for cells to recover overnight in the presence of activating agents (Lipopolysaccharide (LPS* E. coli* O111:B4, EMD Millipore, Billerica, MA, 1 ng/ml) or TNF, 1 ng/ml). The next day, medium was changed to low (2%) FBS medium containing activating agents and vehicle or 50 ng/ml IL-27 and/or 100 ng/ml BMP7 or 10 ng/ml TGF*β*1, and cells were incubated a further 24 h prior to cell collection for luc assay. For T cells, DMRIE-C reagent was used to transfect cells for 6 h; then 20% FBS (Jurkat) or 20% horse serum (EL4) was added to allow cells to recover overnight in the presence of activating agents PMA/I (25 ng/ml phorbol 12-myristate 13-acetate and 500 ng/ml or 1 *μ*M Ionomycin). The next day, medium was changed to low (2%) serum plus or minus IL-27 (50 ng/ml) containing PMA/I or vehicle and cells were incubated a further 24 h prior to cell collection for luc assay. The 96-well white opaque plates (Corning, Corning, NY) were spun at 2000 rpm for 2 min, media were aspirated, and 40 *μ*l 1x passive lysis buffer (Promega, Madison, WI) was added per well and assayed in 96-well format using a Glomax Multi luminometer with luciferin substrate (Luciferase Assay kit, Promega, Madison, WI). 5 *μ*l of the lysates was used to perform a transfection control beta-gal luminescence assay (Clontech) and the remaining 35 *μ*l was used in the luciferase assay following manufacturer's protocols [10].

### 2.4. Real Time Quantitative RT-PCR (qPCR) Analyses

We examined the changes in selected genes associated with osteoblast and osteoclast differentiation using our own primer library. Total RNA from 5–10 × 10^5^ cells was extracted using a SurePrep kit (FisherSci, Pittsburgh, PA). 1-2 *μ*g of RNA was reverse-transcribed using TaqMan (Applied Biosystems, Foster City, CA). One microliter of template cDNA was used in a real time qPCR reaction with 2x SYBR green master mix (Applied Biosystems) and 10 *µ*M of each of forward and reverse primers for experimental or *β*-actin control housekeeping gene. Reactions were run on an Eppendorf Realplex 2S (Hauppauge, NY), using 40x 95°C/15 sec, 56°C/30 sec, and 72°C/30 sec, and analyzed using Realplex software. Primer sequences are available upon request. Results for each assay were normalized to the *β*-actin housekeeping gene expression levels. Data were represented as charts or as heat map histograms.

### 2.5. Statistical Analysis

Assays were performed in triplicate, and values provided as mean ± SEM or 95% confidence interval. Comparisons were performed using an unpaired *t*-test, and *P* < 0.05 was considered to indicate a significant difference.

## 3. Results and Discussion

### 3.1. IL-27 Is an Antagonist of the IL-6/-11 Axis, Promotes Osteoblast Differentiation, and Reduces Osteoclast Formation

**Figure 1:**
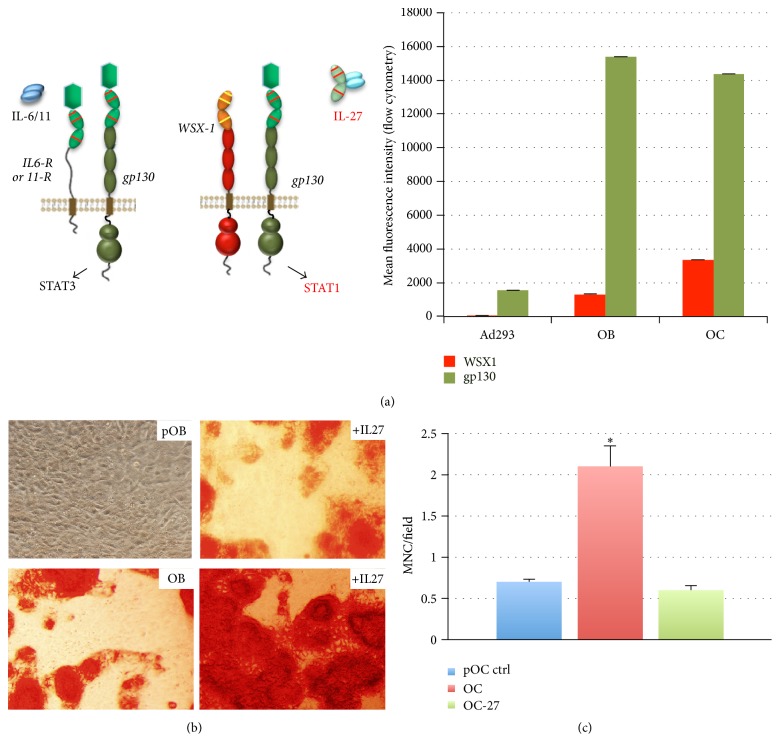
IL-27 promotes osteoblast mineralization and prevents bone erosion. (a) IL-27 is a natural antagonist of IL6/11 and enables cells to coordinate a switch from STAT3 to primarily STAT1-driven cell signaling. Right panel, plot showing expression of receptors WSX1/IL27Ra and gp130 in osteoblasts and osteoclasts in mean fluorescence intensity units as assessed by flow cytometry. (b) Preosteoblast (MC3T3E1 clone 14 or pOB) mineralization as assessed by Alizarin Red staining is enhanced with IL-27 and is further augmented by addition of differentiation supplements containing ascorbic acid and beta-glycerol phosphate. Shown is osteoblastic differentiation by day 21 (OB). (c) Osteoclast (OC) formation from preosteoclasts (pOC) is inhibited by IL-27 (OC-27) even in the presence of RANKL; ^*∗*^*P* < 0.05 osteoclast formation in RANKL+control (OC).

IL-27 is a natural antagonist of the IL-6/11 signaling axis [2], by virtue of competing for binding the gp130 portion of their heterodimeric receptors (Figure 1(a)). IL-27 binding to the receptor WSX1/gp130 enables shifting from a Stat3 to a Stat1 signaling mechanism. We examined the levels of expression of WSX1 and gp130 receptors on MC3T3E1 differentiating osteoblasts (day 7) and RAW264.7-differentiated osteoclasts (day 6) relative to a control cell line (Ad293 or HEK293) by flow cytometry (Figure 1(a), right panel). The expression of these receptors in other cell types such as T cells and NIH3T3 fibroblasts has been shown previously [12, 13]. We examined the ability of IL-27 to promote preosteoblast (pOB) differentiation in the absence of differentiation supplements. IL-27 treatment (+IL-27) induces mineralization in MC3T3E1-14 cultures as compared to untreated control pOB using an Alizarin S Red stain (Figure 1(b)). The addition of differentiation supplements ascorbic acid and beta-glycerol phosphate also induces differentiation, as shown in the osteoblast (OB) control (Figure 1(b)). Addition of IL-27 (+IL-27) in the presence of differentiation supplements further enhanced the extent of mineralization detected by the stain (Figure 1(b)). These results suggested that IL-27 treatment enhances osteoblast differentiation and may have important implications for promoting osteogenesis. These results are in agreement with previous data from our lab whereby in vivo micro-computed tomography analyses showed that mice bearing tibial tumors and treated with a plasmid expressing IL-27 maintained relatively stable bone mass over the course of the study as compared to control plasmid [4].

IL-27 also had an effect in inhibiting osteoclast formation in a cell culture assay for osteoclastogenesis. This assay is based on differentiation of RAW264.7 cells by treatment with RANKL for 6 days. Addition of RANKL to these cell cultures promotes a significant enhancement in cell fusion and osteoclast formation (OC) as compared to control untreated “preosteoblast” cells (pOC) (Figure 1(c)). The treatment of OC cultures with IL-27 prevented the RANKL-mediated osteoclastogenesis (Figure 1(c)), maintaining the multinucleated cell counts to control levels (pOC). This suggested that IL-27 could inhibit RANKL-mediated monocytic cell fusion, reducing numbers of osteoclasts formed in culture. IL-27 thus appears to stimulate osteoblast differentiation under different conditions and also is able to prevent multinucleated osteoclast cell formation; thus IL-27 treatment may protect against bone loss and perhaps promote repair of bone erosions.

### 3.2. IL-27 Coordinates Changes in Expression of Genes Related to Osteoblast Differentiation, Osteoclast Differentiation, and Inflammation

**Figure 2:**
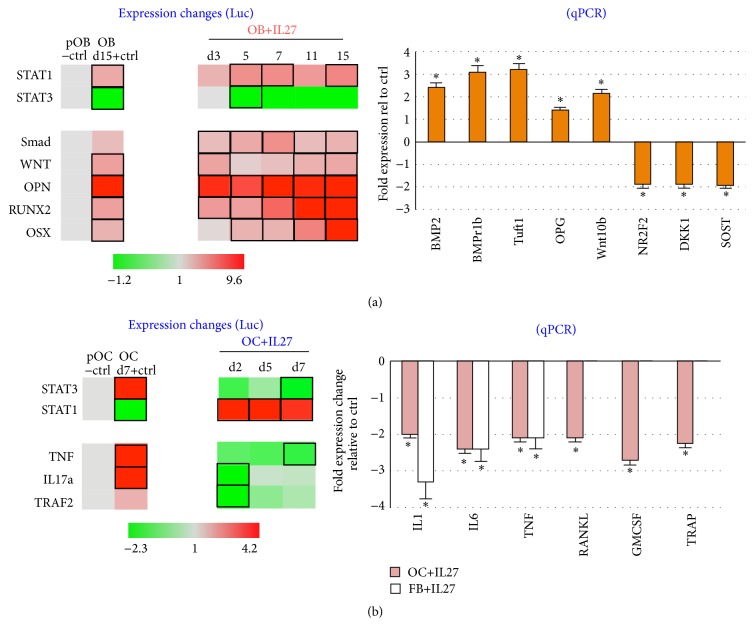
IL-27 effect on osteoblast and osteoclast cell signaling. (a) IL-27 upregulates expression of several genes related to osteoblast differentiation pathways. Expression changes were determined by utilizing a luciferase reporter assay for indicating activity of BMP, WNT, STAT1, STAT3, OPN, RUNX2, and OSX promoters. Black rectangle frames (□) highlight the data with significant expression changes relative to untreated or negative control (pOB−ctrl); +OB ctrl, differentiating MC3T3E1-14 osteoblasts treated with differentiation supplements for 72 h.* Right plot*, IL-27 upregulates osteoblast differentiation genes and downregulates osteoblast inhibitor genes, as assessed by quantitative real time (qPCR) assays. (^*∗*^*P* < 0.05 relative to untreated or negative control, pOB−ctrl). (b) IL-27 downregulates proinflammatory genes in fibroblasts and differentiation genes in osteoclasts, as assessed by quantitative real time (qPCR) assays (^*∗*^*P* < 0.05 versus untreated controls). Right plot, IL-27 treatment downregulates several pathways associated with osteoclast activity as assessed by luciferase reporter assay for indicating activity of TNF, IL-17a, TRAF2, STAT3, and STAT1 promoters. Black rectangle frames (□) highlight the data with significant expression changes (*P* < 0.05) relative to untreated or negative control (pOB−ctrl); +OB ctrl, differentiating MC3T3E1-14 osteoblasts treated with differentiation supplements for the days indicated.

For osteoblasts, we examined the effects on IL-27 treatment on signaling pathways in differentiating MC3T3E1-14 cells over a period of time (days 3 to 15; Figure 2(a)). By using a luciferase reporter assay followed by a heat map representation of the data, we made several findings. First, differentiating osteoblasts treated with IL-27 switch from a predominantly Stat3-driven transcription towards Stat1-mediated signals, and this switch was maintained over the time course examined (Figure 2(a)). Second, relative to control differentiating osteoblasts, IL-27 treatment augmented levels of RUNX2 and OSX over time, while Smad and Wnt signaling were maintained at activated but relatively lower levels. OPN expression was maintained at a relatively high level throughout the differentiation process by IL-27 (OPN levels exceeded 8-fold on some IL-27 timepoints as compared to 4-fold in +Ctrl; Figure 2(a)). Therefore, this luc reporter assay was important to define the markers that are upregulated by IL-27 and the timing associated with early and late markers of differentiation in osteoblasts. The level of expression of early differentiation marker OPN was higher relative to late differentiation markers RUNX2 or OSX, indicating a temporal expression of these genes according to differentiation stage.

Since recent reports suggest that inhibition of osteoblast function may be a key reason underlying bone erosion persistence in conditions such as RA [14], we sought to validate in an independent assay whether IL-27 treatment could modulate not only expression of several genes related to osteogenesis, but also those relating to osteoblast function. Using real time quantitative PCR (qPCR), we observed significant upregulation of BMP2, BMPr1b, Tuft1, OPG, and Wnt10b upon IL-27 treatment in differentiating osteoblasts/osteocytes (^*∗*^*P* < 0.05; Figure 2(a), right plot). This expression signature suggests a role for IL-27 both in promoting a differentiated osteoblast phenotype and potentially in osteogenesis. In addition, we observed downregulation of osteoblast inhibitory genes NR2F2, DKK1, and Sost in IL-27-treated osteoblast/osteocytes (Figure 2(a), right plot), suggesting that IL-27 may enable osteoblasts to escape inhibitory signals from these pathways. Interestingly, these three inhibitory pathways are commonly perturbed in eroded joints in inflammatory arthritis [14].

For osteoclasts, we were also interested in examining potential mechanisms by which IL-27 inhibits RANKL-mediated osteoclast differentiation as shown in our previous work [3, 4]. IL-27 promoted a switch from STAT3- to STAT1-mediated signaling in differentiating osteoclasts in a luciferase reporter assay as compared to positive control (RANKL-treated) osteoclasts (OC, Figure 2(b)). Also, IL-27 reduced the expression of proinflammatory or proosteoclastogenic genes TNF, IL-17a, and TRAF2 in differentiating osteoclasts on days 2, 5, and 7 (Figure 2(b)). Next we expanded the examination of the effect of IL-27 in reducing inflammatory stimuli by examining gene expression changes in osteoclasts or fibroblasts by qPCR. Treatment of RANKL-differentiating osteoclasts (OC) or TNF-activated fibroblasts (FB) with IL-27 resulted in a significant downregulation of several proinflammatory genes including IL-1b, IL-6, and TNF as compared to untreated control OC or FB cells (Figure 2(b), right plot). These results suggested that IL-27 might be able to reduce inflammatory signaling by acting on OC and FB to dampen proinflammatory signaling. Additionally, in osteoclasts, IL-27 treatment downregulated genes associated with osteoclast differentiation including RANKL, GM-CSF, and TRAP, as compared to untreated control OC (Figure 2(b), right plot). Together, these results suggest that IL-27 may inhibit osteoclastogenesis through TRAP downregulation, a net effect likely mediated through impacting two key mechanisms of osteoclast formation in monocytic cells, namely, RANKL and GM-CSF expression.

**Figure 3:**
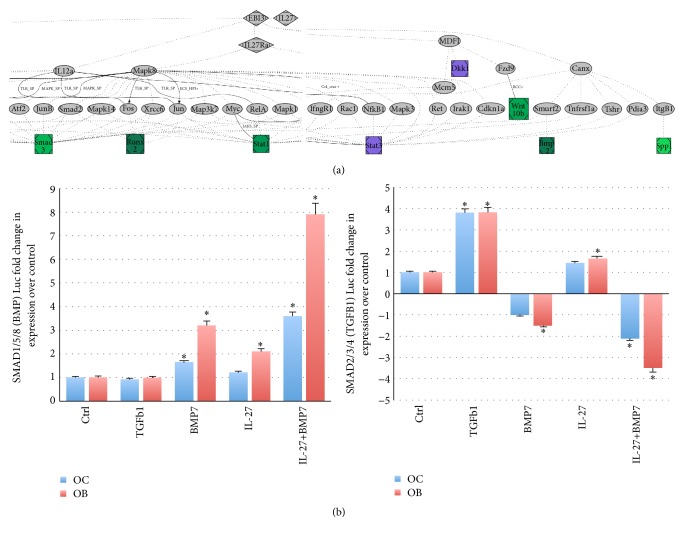
Signaling by IL-27. (a) Example of a PathGen analysis. Output analyses using gene expression and luciferase reporter assay data following IL-27 treatment of osteoblasts. IL-27 is formed by EBI3 and IL27p28 subunits, and it binds to receptor IL27RA (WSX1) with gp130. Image cropped in the center and left edge for summarizing the main signaling connections converging in* Smad3, Runx2, Stat1, and Stat3, and other connections seen to BMP2, SPP1, DKK1,* and* WNT10b*.* Green, upregulated genes; purple, downregulated genes. *(b) Reporter luciferase assay with BRE-Luc (BRE motif binds SMAD1/5/8 and responds to BMP family members). Right plot, reporter luciferase assay with SBE4-Luc (SBE motif binds SMAD2/3/4 and responds to TGFB family members) (^*∗*^*P* < 0.05). OC, differentiating osteoclasts; OB, differentiating osteoblasts.

We performed pathway analysis using the gene expression data for osteoblasts or osteoclasts using PathGen [15], software that uses data from several sources, including KEGG, DIP, and MINT for in silico discovery of novel gene interaction pathways, either between sets of genes or between target genes from a specific biological experiment. These analyses confirmed a connection between the IL-27/IL-27R axis and Stat1 and connected osteogenic genes to IL-27 through Smad3 and osteoblast inhibitory genes to IL-27 through MDFI (MyoD Family Inhibitor), a repressive transcription factor that promotes osteoblast differentiation [16] (Figure 3(a)). Interestingly, previous data from our lab indicated that IL-27 promotes upregulation of ossification gene Tuft1 [3], and other reports have shown that Sost in osteoblasts is regulated via TGF*β*/BMP/Smad [17]. For osteoclast, the connections were made between IL-27 and MDPI through Stat1, suggesting the Smad pathway may be more utilized in osteoblasts to promote expression of maturation genes. Using the data from macrophages and fibroblasts, PathGen linked IL-27 to regulation of IL1*β*, TNF, and IL-6 through the IL12a and NF-*κ*B/Rel pathways and to TRAF2 potentially through MDFI. In most of the cell types examined, it appears that there is a central hub of Smad3/6 downstream of IL-27. Smad transcription factors act not only in bone cells, but also as critical mediators of TGF*β* and BMP anti-inflammatory activity, suppressing IL1R-TLR signaling and preventing NF-*κ*B-mediated expression of proinflammatory genes as well as CSF2 (GM-CSF). And although proinflammatory cytokine IL-17a was not directly connected to the IL-27 network via PathGen, its expression is known to be regulated by IL-27 through reductions in STAT3 activity [18], resulting in reduced TNF and IL-6 proinflammatory gene expression [19].

We have shown that, in response to IL-27 stimulation, several members of the TGF*β*/BMP/SMAD family are upregulated in osteoblasts (SMAD4, BMPR1b, and BMP2), osteoclasts (TGFBR1, BMPR1b), and T cells (IL12p40, BMP2) [3]. Also, IL-12 is upregulated by IL-27 treatment in several cell types in our previous work including T cells [3] and prostate tumors [20]. Functional gene expression assays using luciferase as a reporter gene can also be used to assay differential effects of IL-27 on TGF*β* (SMAD2/3/4; SBE4Luc vector) or BMP (SMAD1/5/8; BRE-Luc vector) pathways (Figure 3(b)). This reporter data suggests the mechanism of IL-27 activity appears to be preferentially mediated through BMP-directed SMAD signaling. Using BRE-Luc, the effect of IL-27 was assessed on SMAD1/5/8 activity in differentiating OC and OB (Figure 3(b)). BMP7 (but not TGF*β*1) and IL-27 each significantly induced SMAD1/5/8 activity. However, when IL-27 was present along with BMP7, SMAD1/5/8 were activated in a synergistic manner in both cell types. Using SBE4-Luc, the effect of IL-27 was assessed on SMAD2/3/4 in differentiating OC and OB (Figure 3(b), right panel). TGF*β*1 (but not BMP7) induced SMAD2/3/4 activation (4-fold), while BMP7 generally inhibited this signaling mode. IL-27 could induce SMAD2/3/4 significantly in OB, although to levels lower than TGF*β*1. The combination of IL-27 and BMP7 acted synergistically to reduce SMAD2/3/4 signaling (Figure 3(b), right panel). One limitation of this data is that it remains to be seen whether IL-27 and TGF*β*1 interact to augment or reduce SBE4-Luc signaling and future studies will examine this interesting hypothesis.

In combination, the prior and current data described above suggested that IL-27 might utilize the TGF*β*/BMP/Smad and/or JAK/Stat1 pathways to promote bone cell balance as well as to potentially reduce inflammatory signaling on other cell types. We therefore postulated that the effect of IL-27 on bone and inflammatory cells could be augmented by adjuvant therapeutic molecules that could regulate the TGF*β*/BMP/Smad or JNK/Stat pathways. Emerging candidate regulators are microRNA mimics or miRNA. Luciferase assay screens were used to compare the effects on signaling of IL-27 treatment alone or in combination with miRNA selected from the literature based on their reported activity in mediating osteogenesis or reversing inflammation in several cell types by modulating these pathways.

### 3.3. miRNA Effects on Signaling in Bone Cells Are Enhanced by Coadministration with IL-27

We selected a panel of miRNA mimics, synthetic double stranded RNA of 20–25 nucleotides that mimic native miRNA functionality in cells. We selected the sequences from the literature based on their reported activity in promoting osteogenesis or anti-inflammatory effects [9]. We utilized either control (nontargeting) or targeting miRNA in the presence or absence of IL-27 coadministration in bone, inflammatory, or immune cells. These cell types were stimulated to model signaling events detectable in cell populations typically involved in the pathogenesis and maintenance of inflammatory arthritis. miRNA effects were examined alone or in combination with IL-27 treatment as changes in gene expression as detected by a luciferase reporter assay. For example, MC3T3-E1(14) stimulated with ascorbic acid and *β*-glycerol-phosphate modeled differentiating osteoblasts, monocytic precursor cells RAW264.7 stimulated with lipopolysaccharide (LPS) modeled proinflammatory macrophages, while RAW264.7 stimulation with RANKL modeled differentiating osteoclasts. Finally, stimulation of NIH3T3 fibroblasts with TNF mimicked fibroblast-like proinflammatory cells, and stimulation of EL4 or Jurkat lymphocytes with phorbol 12-myristate 13-acetate (PMA)/Ionomycin modeled activation of proinflammatory T cells.

**Figure 4:**
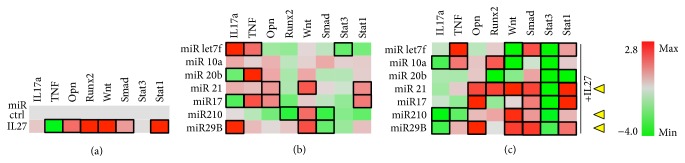
Osteoblastic cells. MC3T3E1(14) cells cultured in the presence of ascorbic acid and beta-glycerol phosphate were cotransfected with miR control or experimental (miRlet7f, miR-10a, miR-20b, miR-21, miR-210, miR-17, and miR-29b) and luciferase reporter vectors for detecting IL17a, TNF, and TRAF2 expression or SMAD2/3, STAT3, and STAT1 activity. (a) Effect of IL-27 (50 ng/ml) on luciferase expression. (b) Effect of experimental miRNA on luciferase expression. (c) Effect of miRNA+IL27 combination on luciferase expression. Black rectangle frames (□) highlight the data with significant expression changes (*P* < 0.05) relative to negative control (miR ctrl); osteoblastic cells, differentiating MC3T3E1-14 cells treated with differentiation supplements for 7 days. Yellow arrowheads, proposed best combinations of miR and IL-27. Color bar, range of fold change in expression, from green (−4.0-fold change or decrease) to red (2.8-fold change or increase).

In differentiating osteoblasts, the luciferase reporter assay showed that IL-27 appeared to reduce TNF expression compared to control transfected with a nontargeting miRNA control (miR ctrl) but without a significant impact on IL-17a, perhaps due to a relatively low induction of the IL-17a transcriptional regulator Stat3, also observed in this assay (Figure 4(a)). In this heat map representation, the statistically significant gene expression changes were highlighted by a black rectangular frame around the appropriate genes. In the same reporter assay, IL-27 upregulated osteoblastic differentiation markers Runx2 and Opn and other pathways related to differentiation, including Wnt/*β*-catenin and TGF*β*/Smad. Finally, IL-27 promoted upregulation of Stat1 by 2.5-fold (Figure 4(a)). For examining the effect of the miRNA alone, we transfected cells with a miR panel and examined the impact on reporter gene expression (Figure 4(b)). miR-20b and 17 significantly downregulated IL-17a but upregulated TNF (Figure 4(b)). Some miRNA (miR) modulated osteoblast differentiation genes, with miR-21 and miR-17 promoting significant upregulation of Opn, for example. Wnt was significantly upregulated by miR-21, miR-210, and miR-29b, while Smad was downregulated by miR-210 and miR-29b. Stat3 was only significantly downregulated by miR-let7f, while miR-21 and miR-17 enhanced Stat1 activity (Figure 4(b)). When examining the effects of combining miRNA and IL-27, there was only one combination that could significantly reduce both IL-17a and TNF signaling, IL-27+miR-210 (Figure 4(c)), although miR-20b and miR-17 also trended towards downregulating these inflammatory signals. IL-27+miR-21 significantly upregulated osteoblast differentiation markers Opn and Runx2, while IL-27+miR-17 or IL-27+29b only upregulated Opn. When combined with IL-27, miR-21, miR-210, and miR-29b upregulated Wnt and Smad, while downregulating Stat3. Stat1 was upregulated when IL-27 was combined with miR-let7f, miR-21, miR-17, or miR-29b. In conclusion, the most beneficial miR for combination with IL-27 in enhancing osteoblast differentiation while suppressing or limiting potential contributions to inflammation appeared to be * miR-21, miR-210, or miR-29b*  (Figure 4(c), yellow arrowheads).

These data are interesting since IL-27 and the miRNAs examined might interact in balancing TGF*β*/BMP/Smad signaling. For example, miR-210 is a positive regulator of osteoblastogenesis that acts by inhibiting the receptor AcvR1*β* [21].* miR-21, miR-210, *and* miR-29b *can each have interactions with IL-27 to switch signaling towards TGF*β*1, which is consistent with enhanced Smad2/3 activity. Potentially, this Smad connection is what amplifies the expression of prodifferentiation genes in osteoblasts. Final examples of miRNA that also could be acting at the TGF*β*/BMP/Smad pathway interface include miR-29b, which increases osteoblast differentiation by repressing the inhibitors of osteogenesis TGF*β*3 and AcvR2*β* [22], and miR-21, which promotes osteogenic differentiation of human mesenchymal/stromal stem cells through Spry-2, a transcription factor that regulates expression of differentiation-associated genes PPARg and Cbfa-1 [23]. Spry-2 connects to both Smad3 and Runx2 through Stat1 and also connects to Opn through PKCa, according to our PathGen analyses. Therefore, the effect of IL-27 and miR on OB appears to operate through Smad2/3 and Stat1 signaling.

**Figure 5:**
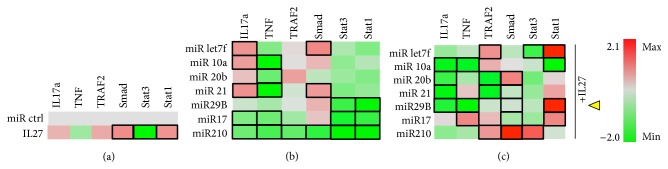
Osteoclastic cells. RAW264.7 cells cultured in the presence of 35 ng/ml RANKL and cotransfected with miR control or experimental (miRlet7f, miR-10a, miR-20b, miR-21, miR-29b, miR-17, and miR-210) and luciferase reporter vectors for detecting IL17a, TNF, and TRAF2 expression or SMAD2/3, STAT3, and STAT1 activity. (a) Effect of IL-27 (50 ng/ml) on luciferase expression. (b) Effect of experimental miRNA on luciferase expression. (c) Effect of miRNA+IL27 combination on luciferase expression. Black rectangle frames (□) highlight the data with significant expression changes (*P* < 0.05) relative to negative control (miR ctrl); osteoclasts, differentiating RAW264.7 cells treated with differentiation supplements for 6 days. Yellow arrowheads, proposed best combinations of miR and IL-27. Color bar, range of fold change in expression, from green (−2.0-fold change or decrease) to red (2.1-fold change or increase).

In differentiating osteoclasts, IL-27 treatment appeared to enhance Smad and Stat1 and reduce Stat3 significantly. There was a trend towards increased IL-17a and TRAF2, but these changes were not significant (Figure 5(a)). miR-17 and miR-210 were the only ones able to significantly downregulate IL-17a and TNF in differentiating osteoclasts and all miR downregulated TNF and Stat3 to some extent without upregulating Stat1. miR-let7f and miR-21 were the only ones that significantly upregulated Smad (Figure 4(b)). Combination of IL-27 and either miR-10a or miR-29b appeared to significantly reduce TNF and IL-17a, whereas IL-27+miR-20b or +21 promoted significant downregulation only of IL-17a (Figure 4(c)). TRAF2, a TNFR/RANK signaling pathway component, was upregulated by IL-27+miR-let7f or +210. These miR could potentially be detrimental to the IL-27 effects on osteoclastogenesis since RANKL signaling recruits TRAF adaptor proteins to RANK during osteoclast differentiation [24]. Two miR combinations maintained (miR-20b) or augmented (miR-210) Smad activation by IL-27 in osteoclasts (Figure 5(c)). Stat1 activity was augmented when IL-27 was combined with miR-let7f, miR-29b, or miR-17 (Figure 5(c)). Based on these findings, the miR that most likely would assist IL-27 in reducing osteoclastogenesis would be * miR-29b* (Figure 5(c), yellow arrowhead), as it significantly reduced IL-17a and TNF, maintained STAT3 inhibition, and upregulated STAT1, although* miR-let7f*,* miR*-*20b*, and* miR*-*210* also could be useful as they shared some similar trends in altered proinflammatory signaling. miR-29b is interesting for complementing the effect of IL-27 in inhibiting osteoclast differentiation since it targets the c-Fos/NFATc1/TRAP signaling axis [25], while IL-27 inhibits both RANKL- and TNF-mediated signaling [3]. When combined, miR-29b and IL-27 might inhibit a broader range of signaling cascades, including TNF, RANK/TRAFs, and RANK/c-Fos, reducing TRAP and expression of other osteoclast-specific genes. The overall effect of IL-27 and miR-29b in TNF and TRAF signaling appears to be less dependent on Smad activation and correlates more with Stat1 activation.

### 3.4. miRNA Effects on Signaling in Inflammatory Cells Are Enhanced by Coadministration with IL-27

**Figure 6:**
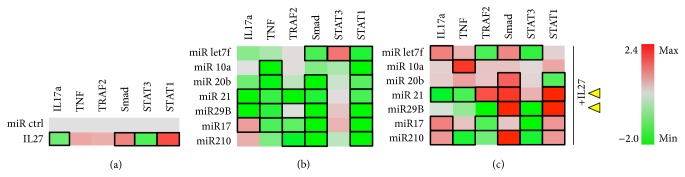
Macrophage cells. RAW264.7 macrophages cultured in the presence of proinflammatory LPS stimulus and cotransfected with miR control or experimental (miRlet7f, miR-10a, miR-20b, miR-21, miR-29b, miR-17, and miR-210) and luciferase reporter vectors for detecting IL17a, TNF, and TRAF2 expression or SMAD2/3, STAT3, and STAT1 activity. (a) Effect of IL-27 (50 ng/ml) on luciferase expression. (b) Effect of experimental miRNA on luciferase expression. (c) Effect of miRNA+IL27 combination on luciferase expression. Black rectangle frames (□) highlight the data with significant expression changes (*P* < 0.05) relative to negative control (miR ctrl); macrophages, RAW264.7 cells treated with activating agent LPS as per Materials and Methods. Yellow arrowheads, proposed best combinations of miR and IL-27. Color bar, range of fold change in expression, from green (−2.0-fold change or decrease) to red (2.4-fold change or increase).

In cells of a monocytic lineage stimulated with LPS to mimic proinflammatory macrophage activation, IL-27 upregulated Stat1 and Smad while downregulating IL-17a and Stat3 significantly (Figure 6(a)). Minor increases in TNF and TRAF2 observed were not significant compared to control. For miRNA, miR-21, and miR-29b promoted significant downregulation of both IL-17a and TNF (Figure 6(b)). Smad was downregulated by the majority of miR, whereas only miR-let7f upregulated Stat3 significantly, an effect that could be detrimental in the context of inflammation. Other miR were tested including miR29b, miR210, and miR17, and these showed no significant differences (data not shown). Combination of IL-27+miR-21 was promising for therapeutic applications in that it reduced IL-17a and TNF while upregulating Stat1 activity (Figure 6(c)). miR-29b also complemented IL-27, since it did not significantly worsen IL-17a signaling compared to miR control, yet it reduced TNF and Stat3 activity and upregulated Smad and Stat1 to levels 1.5-fold higher than IL-27 alone. Thus, according to these data, the most beneficial miR to combine with IL-27 for reducing inflammatory activity of macrophages would be * miR-21 and miR-29b*  (Figure 6(c), yellow arrowheads), as they downregulate or stabilize IL-17a levels and downregulate TNF and Stat3, while enhancing the effect of IL-27 on Stat1 activation. The rebalancing of Stat3-to-Stat1 signaling in the context of IL-17a or TNF downregulation may promote an anti-inflammatory environment and be beneficial therapeutically, as Stat3 has been linked to regulation of NF-*κ*B- and IL-6-related proinflammatory mechanisms in RA [26]. miR-21 has been associated with an anti-inflammatory role in macrophages by multiple mechanisms including reducing TNF secretion and inhibiting NF-*κ*B and IL-6 production during LPS stimulation [27]. miR-29b could help modulate anti-inflammatory signals through its inhibitory role in both TGF*β*3/AcvR2*β* and NF-*κ*B pathways [28], although since increases in miR-29b levels appear to follow some macrophage LPS stimulation protocols [29], it remains unknown whether this miRNA may be part of an early anti-inflammatory response or whether it may promote inflammation.

**Figure 7:**
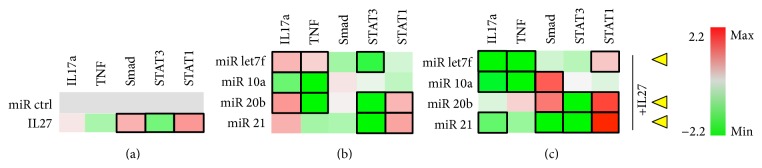
Fibroblastic cells. NIH 3T3 fibroblastic cells grown in the presence of TNF stimulus and cotransfected with miR control or experimental (miRlet7f, miR-10a, miR-20b, or miR-21) and luciferase reporter vectors for detecting IL17a and TNF expression or STAT3 and STAT1 activity. (a) Effect of IL-27 (50 ng/ml) on luciferase expression. (b) Effect of experimental miRNA on luciferase expression. (c) Effect of miRNA+IL27 combination on luciferase expression. Black rectangle frames (□) highlight the data with significant expression changes (*P* < 0.05) relative to negative control (miR ctrl); fibroblastic cells, NIH3T3 cells treated with activating agent TNF as per Materials and Methods. Yellow arrowheads, proposed best combinations of miR and IL-27. Color bar, range of fold change in expression, from green (−2.2-fold change or decrease) to red (2.2-fold change or increase).

In fibroblastic cells stimulated with TNF to induce proinflammatory signaling, IL-27 treatment did not significantly alter IL-17a or TNF signaling, yet it induced Smad and Stat1 signaling and reduced Stat3 (Figure 7(a)). For miRNA, only miR-10a significantly reduced IL-17a expression (Figure 7(b)). miR10a and 20b both significantly reduced expression of TNF, and miR-let7f and miR-21 also reduced Smad signaling; however this difference was not significant (Figure 7(b)). miR-20b and miR-21 both induced Stat1 activity while reducing Stat3 (Figure 7(b)). Other miR were tested including miR29b, miR210, and miR17, and these showed no significant differences (data not shown). When IL-27 was coadministered, miR-let7f, miR-10a, and miR-21 were able to significantly downregulate IL-17a, whereas miR-letf7f and miR-10a also could downregulate TNF, and miR-20b and miR-21 could reduce Stat3 and augment Stat1 activation (Figure 7(c)). These effects on Stat3-to-Stat1 switching, as discussed above, can be beneficial for reducing proinflammatory signaling. Also, miR-10a and 20b could enhance Smad activation when in combination with IL-27 (Figure 7(c)). Therefore, when combined with IL-27,* miR-21, miR-let7f, or miR-20b* appear to be the most promising for reducing inflammatory signaling while enhancing Stat1 in fibroblastic cells (Figure 7(c), yellow arrowheads). Of these, perhaps * miR-20b*  is the most promising for use with IL-27, since it has a role in inhibiting inflammatory processes in mouse models of asthma through reducing expression of VEGF [30]. Although miR-21 might have a proinflammatory effect, as shown for inflammatory bowel disease [31], in combination with IL-27 it appears to augment some of its signaling effects. miR-let7f targets A20, a feedback inhibitor of the NF-*κ*B pathway, but may also enhance production of cytokines TNF and IL1*β* under certain infectious conditions [32].

### 3.5. miRNA Effects on Signaling in Immune Cells Are Enhanced by Coadministration with IL-27

**Figure 8:**
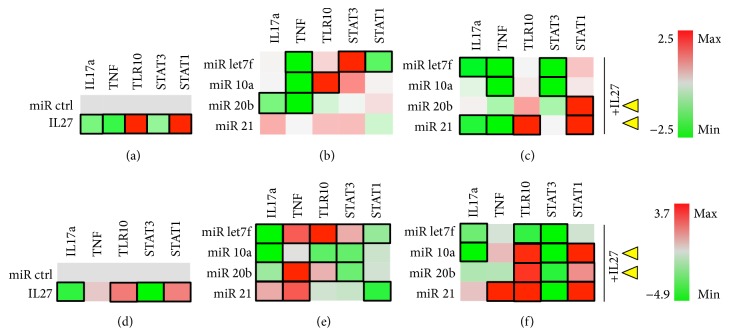
Jurkat T cells. T cells were grown in the absence (a–c) or presence (d–f) of PMA/Ionomycin stimulus. Cells were cotransfected with miR control or experimental (miRlet7f, miR-10a, miR-20b, or miR-21) and luciferase reporter vectors for detecting IL17a, TNF, and TLR10 expression or STAT3 and STAT1 activity. (a) Effect of IL-27 (50 ng/ml) on luciferase expression. (b) Effect of experimental miRNA on luciferase expression. (c) Effect of miRNA+IL27 combination on luciferase expression. Black rectangle frames (□) highlight the data with significant expression changes (*P* < 0.05) relative to negative control (miR ctrl); Jurkat T cells cultured in the absence (a–c) or presence of PMA/I (d–f) as per Materials and Methods. Yellow arrowheads, proposed best combinations of miR and IL-27. Color bars, range of fold change in expression, from green (−2.4- or −4.9-fold change or decrease) to red (2.5- or 3.7-fold change or increase).

For lymphocytic cells, we examined both mouse (EL4) and human (Jurkat) T cell lines. In Jurkat T cells, IL-27 addition promoted downregulation of IL17a, TNF, and STAT3 and upregulation of TLR10 and STAT1 (Figure 8(a)). For miRNA, IL-17a expression was downregulated only by miR-20b, whereas TNF was downregulated by miR-let7f, miR-20b, and miR-10a (Figure 8(b)). Significant stimulation of TLR10 expression only was seen with miR-10a, and expression of TLR10 can be used as a marker of regulatory T cells expressing transcription factor FoxP3/NF-AT [33]. Stat3 activity was significantly stimulated and Stat1 activity was reduced by miR-let7f (Figure 8(b)). Other miR were tested including miR29b, miR210, and miR17, and these showed no significant differences (data not shown). IL-27, when coadministered with miR-let7f and miR-21 significantly downregulated IL-17a and TNF, while only the IL27+miR21 combination upregulated TLR10 and Stat1 signals (Figure 8(c)). IL-27 enhances the effects of miR-let7f in downregulating TNF by 10% and of miR-21 by 40%, while maintaining the TNF suppression effects of miR-10a and miR-20b. IL-27 enhanced the effects of miR-21 on downregulating IL-17a and maintained the effects of the other miRNAs. IL-27 also significantly enhanced the effect of miR-21 on TLR10 upregulation (also of miR-20b but the difference was not significant). IL-27 works to suppress Stat3 activity in most of the miR-treated samples, while upregulating Stat1 in most samples, but especially when in combination with miR-20b and miR-21. In sum, the combination of IL-27 and * miR-21* is the most promising for upregulating TLR10 while downregulating IL-17a and TNF, with * miR-20b,*  a potential candidate as well.

In the presence of PMA/I stimulation, the effects of IL-27 do not seem to be greatly altered (Figure 8(d)), and miRNAs are generally similar, except that miR-let7f appears to augment TNF and TLR10 and miR-20b and miR-21 appear to upregulate TNF and/or IL-17a (Figure 8(e)). The combination of miR+IL27 was different in the presence of PMA/I and appeared to augment TLR10 activation by IL-27 in combination with miR-10a, miR-20b, and miR-21, with corresponding Stat3 downregulation and Stat1 upregulation (Figure 8(f)). Interestingly, PMA/I stimulation contributed to miR-21 upregulation of TNF, even when present in combination with IL-27. This suggests the miR-21 combination with IL-27 might be most effective at earlier stages of T cell activation, that is, early inflammatory processes, and perhaps the miR-20b+IL27 combination could be more useful for later inflammatory processes following T cell activation.

**Figure 9:**
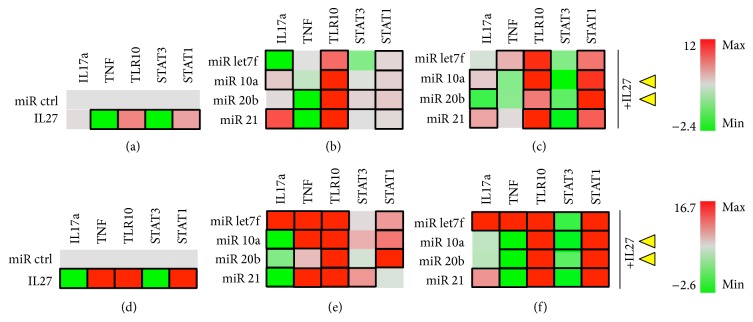
EL4 T cells. T cells were grown in the absence (a–c) or presence (d–f) of PMA/Ionomycin stimulus. Cells were cotransfected with miR control or experimental (miRlet7f, miR-10a, miR-20b, or miR-21) and luciferase reporter vectors for detecting IL17a, TNF, and TLR10 expression or STAT3 and STAT1 activity. (a) Effect of IL-27 (50 ng/ml) on luciferase expression. (b) Effect of experimental miRNA on luciferase expression. (c) Effect of miRNA+IL27 combination on luciferase expression. Black rectangle frames (□) highlight the data with significant expression changes (*P* < 0.05) relative to negative control (miR ctrl); EL4 T cells cultured in the absence (a–c) or presence of PMA/I (d–f) as per Materials and Methods. Yellow arrowheads, proposed best combinations of miR and IL-27. Color bar, range of fold change in expression, from green (−2.4- or −2.6-fold change or decrease) to red (12.0- or 16.7-fold change or increase).

In mouse EL4 T cells, generally similar effects were observed with miR and IL-27. IL-27 significantly downregulated TNF and Stat3 while upregulating TLR10 and Stat1 (Figure 9(a)). For miRNA, IL-17a was downregulated by miR-let7f and upregulated by miR-10a and miR-21, while TNF was downregulated by miR-20b and miR-21 (Figure 9(b)). TLR10 was upregulated by all miR tested, with the highest magnitude from miR-10a and miR-21 treatment (>8.0-fold; Figure 9(b)). miR-let7f reduced Stat3 (although not significantly) and upregulated Stat1 activity. Also, miR-10a, miR-20b, and miR-21 upregulated Stat1 activity significantly (Figure 9(b)). In combination with miR-20b, IL-27 promoted downregulation of IL-17a (Figure 9(c)), and IL-27 attenuated the effect of miR-21 on IL-17a upregulation. However, when IL-27 was combined with miR-let7f, this could result in a detrimental effect due to upregulation of TNF. IL-27 maintained the effects of miR-10a and miR-20b on downregulating TNF and augmented the effects of miR-10a on TLR10 upregulation by 33% (from 8-fold to 12-fold; Figure 9(c)). IL-27 downregulated Stat3 and upregulated Stat1 in combination with all miRNA examined, but significantly with * miR-20b*  (Figure 9(c)).

PMA/I stimulation appeared to induce some detrimental modifications on how IL-27 signals in the presence of miRNA, with an enhanced upregulation of IL-17a and TNF by miR-let7f and some reduction in the magnitude of TLR10 induction (~25–60%) for all miR. However, we saw upregulation of IL-17a when IL-27 was combined with let7f or miR21 and upregulation of TNF when IL-27 was either alone or combined with let7f, miR10a, or miR21 (Figures 9(e) and 9(f)). Further upregulation of TLR10 was seen when IL-27 was combined with miR-10a or miR-21 in the presence of PMA/I (Figure 9(f)). Therefore, IL-27 might best be combined with * miR-10a or miR-20b*, considering the high TLR10 stimulation achieved, but while achieving an insignificant activation of IL-17a and TNF, even in the presence of strong PMA/I stimuli. Again, the choice of combination of IL-27 with miRNA might vary depending on the stage of T cell activation examined, and perhaps early inflammatory responses would be favored therapeutically.

## 4. Conclusions

We have shown that IL-27 promotes osteoblast differentiation, reduces expression of osteoblast inhibitory genes, and reduces osteoclast differentiation, and results suggest a potential coordination with TGF*β*/BMP pathways. We selected miRNA regulators of these and related pathways to examine whether the effects of IL-27 could be augmented for therapeutic applications and observed that miR-29b and miR-21 augmented IL-27 proosteogenic while downregulating osteoclastogenic signals. The same miRNA also worked to reduce inflammatory signaling in activated macrophages, while miR-21 and miR-20b worked with IL-27 to reduce inflammatory gene expression in fibroblasts and T cells. It thus appears that miRNA can be utilized to enhance IL-27's impact on modulating osteogenesis and reducing proinflammatory signaling, with miR-29b, miR-21, and miR-20b holding the most promise across all the miRNAs tested. A limitation of our study is that we utilized immortalized cell lines treated with the miRNA or cytokines studied, and our goal for future studies is to validate these findings in primary mouse and human cells from healthy or RA patients, to promote potential translatability to the clinic.
